# Beta-lactamase-negative ampicillin-resistant *Haemophilus influenzae* type b meningitis in partially immunized immunocompetent child: a case report

**DOI:** 10.1186/s13256-021-03041-8

**Published:** 2021-08-18

**Authors:** Majid Ali Qureshi, Imran Asad, Adeel Chaudhary, Walid Abuhammour

**Affiliations:** 1 Mohammed Bin Rashid University of Medicine and Health Sciences (MBRU), Al Jaddaf, Dubai, United Arab Emirates; 2 Paediatric Emergency Department, Al Jalila Children’s Specialty Hospital Al Jaddaf, Dubai, United Arab Emirates; 3 Paediatric Infectious Disease Department, Al Jalila Children’s Specialty Hospital, Dubai, United Arab Emirates

**Keywords:** Emergency medicine, Pediatrics (drugs and medicines) < drugs and medicines, Meningitis < infectious diseases, Pediatric intensive care < intensive care, Infection (neurology) < neurology

## Abstract

**Introduction:**

*Haemophilus influenzae* is a Gram-negative coccobacillus that can cause many different kinds of infection, ranging from mild ear infection to life-threatening diseases like epiglottitis and meningitis. Encapsulated type b *Haemophilus influenzae* was most commonly responsible for *Haemophilus influenzae* meningitis in children before introduction of *Haemophilus influenzae* conjugate vaccine. None or partially immunized children are acquiring meningitis owing to resistant strains of *Haemophilus influenzae*, namely beta-lactamase-negative ampicillin-resistant strain.

**Case presentation:**

We reported the case of a 2-year-old Emirati boy who presented to our emergency department with fever, diarrhea, vomiting, and fluctuating levels of consciousness. He was developmentally normal with no significant past medical history, except he was partially immunized. Earlier, he had been treated for acute gastroenteritis with intravenous fluids and antiemetics in another hospital and was discharged. His parents escorted him to our emergency department as he became very drowsy. Examination revealed that he was in septic shock. He was immediately treated with oxygen, intravenous antibiotics, and fluids after performing septic workup. He was then shifted to intensive care unit. Blood culture and cerebrospinal fluid Gram stain confirmed diagnosis of beta-lactamase-negative ampicillin-resistant *Haemophilus influenzae*. He was started on intravenous ceftriaxone, acyclovir, and dexamethasone. He still spiked fever after 1 week. Therefore, ceftriaxone was replaced by meropenem. He recovered well with no sequelae.

**Conclusion:**

This case highlights atypical presentation of life-threatening illness along with microbial resistance that had positive outcome due to timely diagnosis and aggressive management by a multidisciplinary team.

## Introduction

According to immunization program in the United Arab Emirates (UAE), *Haemophilus influenzae* (Hib) vaccine is given at age of 2, 4, and 6 months, with a booster dose at the age of 18 months [1]. Encapsulated type b *Haemophilus influenzae* was the commonest cause of meningitis especially in children under the age of 5 years in the UAE before introduction of Hib conjugate vaccine in 1999 [2]. This life-threatening condition can have atypical presentation impersonating a simple viral illness. If not diagnosed and treated promptly, it can result in high morbidity and mortality. High index of clinical suspicion is required to make a diagnosis in these children. Careful monitoring of neurological functions [neuro-observations/magnetic resonance imaging (MRI) and so on] is required to detect complications (deafness, nerve palsy, brain damage) early. Henceforth, a multidisciplinary team approach is the best way forward for the management of these children. Partially immunized children are developing resistant strains of *Haemophilus influenzae* such as beta-lactamase-negative ampicillin-resistant (BLNAR) strain. These strains are frequently causing meningitis in children (children under age of 5 years are at high risk) in the UAE [2]. These strains are more responsive to combination of ceftriaxone and meropenem but are resistant to amoxicillin–clavulanic acid and most of the cephalosporins.

## Case presentation

### Patient information

A previously healthy 2-year-old Emirati boy presented to our emergency department (ED) with a history of cold, cough, and runny nose of 1-week duration. He developed fever of 39.5 °C at home a day prior to presentation responding to antipyretics. Earlier on the day of presentation, he vomited four times at home along with two episodes of loose stools. His parents took him to another ED, where he was treated with antiemetics and intravenous fluids for acute viral gastroenteritis. He was discharged in the late afternoon. On his way back, he vomited once again and appeared very drowsy. He was then brought to our ED. According to the parents, he was alert and responsive throughout the day. They also denied any neck pain, neck stiffness, rash, or photophobia. By the time, he arrived at ED, he had fluctuating levels of consciousness and fever. Most importantly, his parents admitted that he did not receive any immunizations after the age of 4 months. There was no history of sick contacts, or any recent travel. There was no significant past medical, surgical, or family history.

### Clinical findings

On examination, he was drowsy but arousable [Glasgow Coma Scale (GCS) fluctuating between 10 and 11], and had low-grade fever (38 °C), tachycardia (137 beats/minute), hypotension (80/50 mmHg), and capillary refill time of 4 seconds. Both pupils were of size 3 with slow reaction to light and accommodation. He had no neck stiffness or any signs of meningeal irritation. All other systematic examination was normal except ear, nose, and throat, which revealed erythematous mildly enlarged tonsils.

### Timeline

**Fig. 1:**
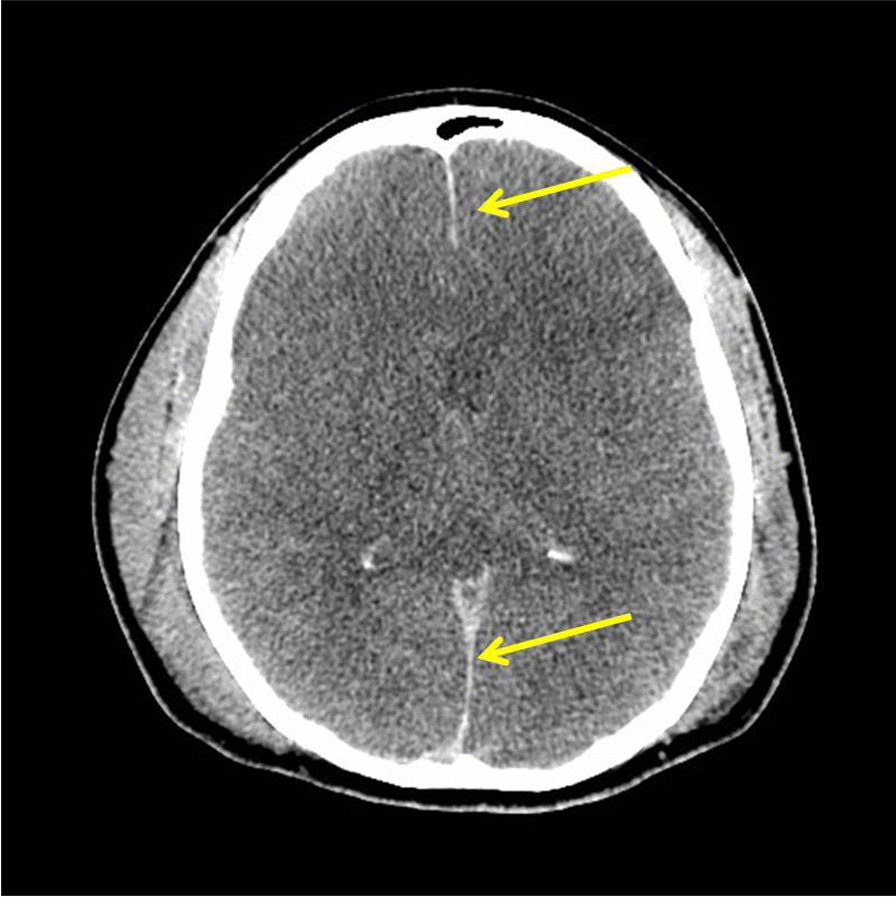
Computerized tomography (CT) head - arrows pointing towards diffuse cerebral edema

Due to presentation of child with fever and fluctuating level accompanied with raised capillary refill time (CRT), hypotension, and tachycardia, a diagnosis of septic shock secondary to meningitis and encephalitis was highly suspected. He was promptly treated with intravenous ceftriaxone, acyclovir, dexamethasone, and fluids within the first hour of his arrival in ED. Computed tomography (CT) head was performed immediately after the administration of antibiotics, which revealed mild diffuse cerebral edema with no signs of raised intracranial pressure (Fig. 1).

**Fig. 2:**
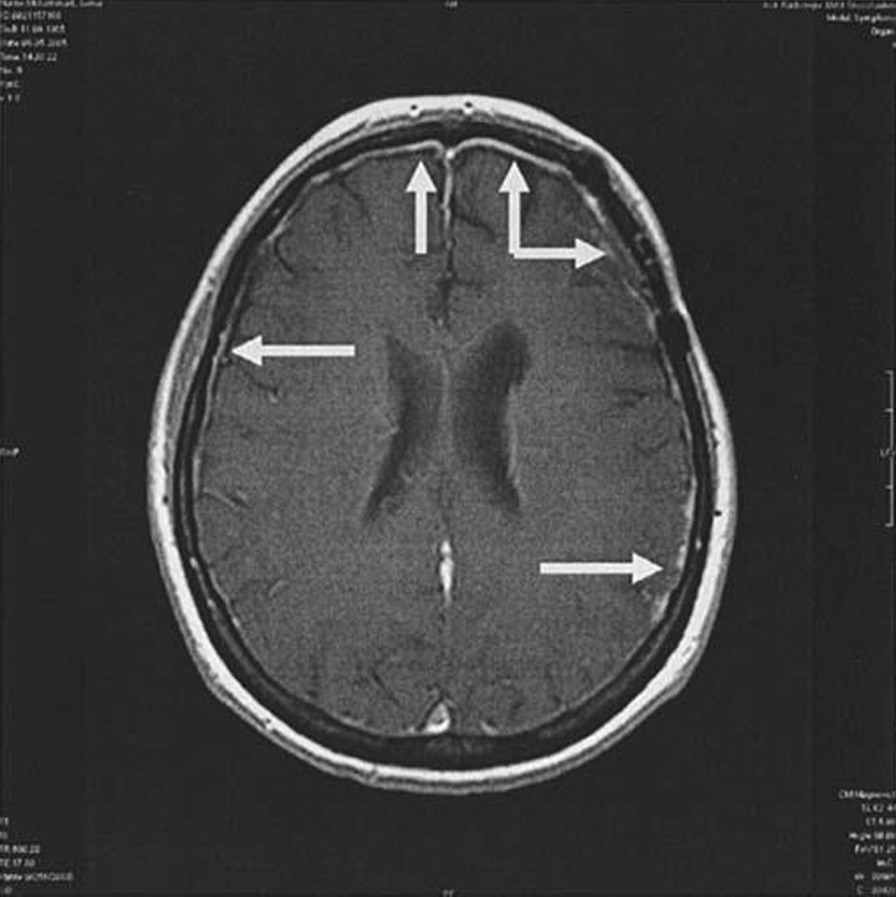
Magnetic resonance imaging (MRI) head - arrows pointing towards meningeal enhancement

Initially, he was admitted to pediatric intensive care unit (PICU) for 6 days before being moved to general ward. CSF and blood cultures confirmed beta-lactamase-negative ampicillin-resistant (BLNAR) *Haemophilus influenzae* type b meningitis susceptible to ceftriaxone, meropenem, and cefotaxime, so it was decided to continue with intravenous ceftriaxone (200 mg/kg/day) and dexamethasone. First MRI (on day 2) depicted meningeal enhancement consistent with meningitis (Fig. 2).

**Fig. 3:**
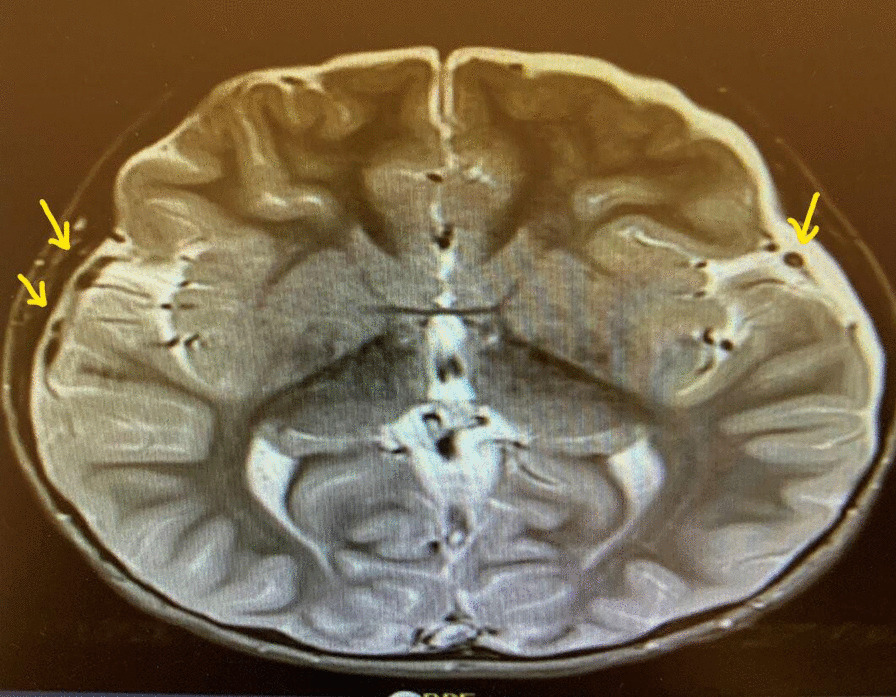
Magnetic resonance imaging (MRI) head - arrows pointing towards pockets of pus

**Fig. 4:**
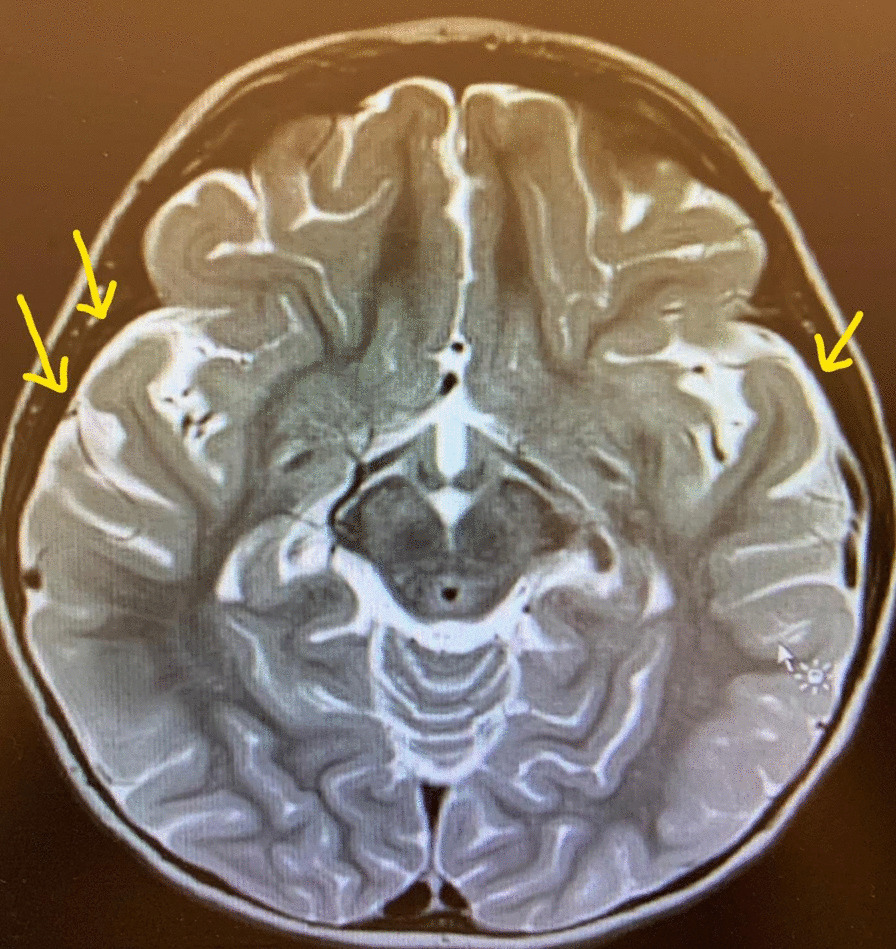
Magnetic resonance imaging (MRI) head - arrows pointing towards resolution of pockets of pus

As the child was still spiking fever on day 7, second blood culture was obtained, which manifested BLNAR strain sensitive to meropenem and resistance to ceftriaxone; hence, antibiotic was changed to meropenem (120 mg/kg/day). He responded well to change of antibiotic and became afebrile. Owing to continuous irritability of the child, a second MRI was performed on day 8 of admission, which demonstrated small pockets of pus in subdural area, for which neurosurgical opinion was sought (Fig. 3). Neurosurgeon advised to carry on with intravenous antibiotics and repeat MRI in 1 week’s time (or earlier in the case of further deterioration). A follow-up MRI (prior to discharge) was reassuring as it confirmed resolution of small pockets of pus (Fig. 4).

Antibiotics were given for 14 days after consultation with infectious disease team. All blood investigations (including infection markers and blood cultures) and hearing tests performed before discharge were normal. The child was discharged on day 17 after satisfactory recovery and no immediate complications.

## Diagnostic assessments

Investigations were performed to determine focus of infection. Initial blood test results in emergency department were:

- White blood cells (WBC)—2.73  (**low**)
- Neutrophil %—80.20 (**high**)
- C-Reactive protein (CRP)—72.91 mg/L (**high**)
- Procalcitonin—95.72 ng/ml (**very high**)
- Prothrombin time—19.4 seconds (**high**)
- Partial thromboplastin time—41.2 seconds (**high**)
- International normalized ratio—1.65 (**high**)
- Lactate—3 mmol/L (**high**)
- First blood culture (on admission)—BLNAR strain of Haemophilus influenzae (indeterminate strains with MIC ≥ 2 excluded) sensitive to ceftriaxone and meropenem resistant to ampicillin/clavulanic acid isolated after 24 hours

**Table:** 

Antibiotic	Concentrations and method	Sensitive/resistant
Amoxicillin/clavulanate	10 μg/ml (MIC)	Resistant
Ampicillin	7.4 μg/ml (MIC)	Resistant
Cefotaxime	0.08 μg/ml (ETEST)	Sensitive
Ceftriaxone	0.02 μg/ml (ETEST)	Sensitive
Meropenem	16–32 mm (disk diffusion)	Sensitive
- CT scan (on admission)—mild diffuse cerebral edema (Fig. 1)
- Delayed LP (day 2 after correcting deranged clotting screen) results:
  - CSF protein—105 mg/dl (**high**)
  - CSF glucose—35 mg/dl (**low**) blood glucose—80 mg/dl (**normal**) CSF glucose/blood glucose—0.4 (**low**)
  - WBC cerebrospinal fluid (CSF)—5566 (**very high**)
  - Polys CSF—89 (**high**)
  - Gram stain—Gram-negative coccobacilli
- Second blood culture (day 7)—BLNAR strain sensitive to meropenem and cefuroxime, resistant to ceftriaxone and ampicillin/clavulanic acid
- MRI brain (day 2 post admission)—meningeal enhancement consistent with meningitis (Fig. 2)
- MRI brain (day 8 post admission)—small pockets of pus—subdural effusion (Fig. 3)
- Blood culture (day 15 post admission)—no growth
- WBC/procalcitonin/CRP (day 15 post admission)—within normal range
- MRI brain (day 16 post admission)—resolution of small pockets of pus (Fig. 4)
- Typing of Haemophilus influenza—type b
- Immunoglobulins (IgA, IgM, IgG)—within normal range

## Differential diagnosis

A diagnosis of septic shock (tachycardia, hypotension, reduced conscious level, and increased CRT) was made while considering a wide range of differentials, including meningitis, encephalitis, postictal phase (secondary to febrile convulsion), raised intracranial pressure due to mass lesion, pneumonia, urinary tract infection, or severe dehydration (secondary to vomiting with no oral intake).

## Therapeutic interventions

The child received intravenous ceftriaxone (100 mg/kg) and fluid bolus within 60 minutes of his arrival to ED. He was also given intravenous acyclovir to cover for encephalitis. In ICU, he was looked after by a team of intensivist, neurologist and infectious disease specialist. Due to persistent tachycardia, hypotension, and raised CRT (despite administration of two normal saline boluses and on full intravenous maintenance), he was kept on intravenous dopamine for 1 day. After confirmation of *Haemophilus influenzae* meningitis (BLNAR strain) with blood culture, it was decided to start him on high dose of ceftriaxone (200 mg/kg/day) for 10 days along with intravenous dexamethasone for 4 days. Acyclovir was stopped. He was also given fresh frozen plasma and vitamin K to stabilize his deranged clotting screen and high INR before performing LP. After 6 days in ICU, he was shifted to general ward.

In ward, he still kept spiking fever (> 39 °C) while on high dose of intravenous ceftriaxone for 7 days. After repeat blood culture (which confirmed BLNAR strain resistant to ceftriaxone), antibiotic was changed to meropenem (120 mg/kg/day) with good results as he became afebrile. This new antibiotic was continued for 14 days.

## Follow-up and outcomes

The child had an excellent outcome and was discharged without any immediate sequela or complications of *Haemophilus influenzae* meningitis. He was then followed up with neurology and infectious disease team 2 weeks after discharge with no issues identified yet. A further follow-up at 6 months was also satisfactory. He achieved all his developmental milestones appropriate to his age after discharge (like beginning to run, standing on tiptoe, building tower of four blocks or more, using two-to-four-word sentences and beginning to sort by shapes or colors). He was also booked in vaccination clinic after discharge to complete his vaccinations as per UAE immunization schedule (from fourth month onward).

## Discussion

Here we report a case of meningitis caused by BLNAR strain of *Haemophilus influenzae* type b (Hib) resistant to ceftriaxone and sensitive to meropenem in a 2-year-old boy, who has recovered completely with no immediate sequelae or complications. Review of literature yielded eight articles, in which total five cases were reported.

There was only one case reported about an infant in which meropenem was effective against bacterial meningitis due to BLNAR strain of *Haemophilus influenzae* compared with other antibacterial drugs [3]. Two case reports are about failure of second- and third-generation cephalosporins in treatment of bacterial meningitis (due to BLNAR strain of Hib). Out of these two case reports, one case report is about three children with meningitis due to Hib, who were treated with high dose of cefamandole (200 mg/kg/day), including one child with disease due to ampicillin-resistant strain [4]. All patients showed clinical improvement during therapy. However, sterility of CSF was never achieved in two patients during 72–96 hours of therapy with cefamandole. The third patient relapsed with a recurrence of positive cultures during the seventh day of cefamandole. Therefore, case report proved that cefamandole does not appear to be a useful agent for treatment of meningitis due to Hib. Another case report is about a 10-year-old boy, who presented with nuchal rigidity and CSF leukocytosis initially and again on day 6 of intravenous cefuroxime therapy (200 mg/kg/day) [5]. Both CSF specimens yielded nontypeable beta-lactamase-negative *Haemophilus influenzae* that was susceptible by disk tests but relatively resistant to cefuroxime. Another case report is about a 1-year old girl with bacterial meningitis due to nontypeable *Haemophilus influenzae* (NTHi), with no significant medical history [6]. Some beta-lactams were administered, but fever was prolonged. Finally, rifampicin seemed to be effective in NTHi. The last case report is about a 1-year old Japanese boy, who developed meningitis due to BLNAR strain of Hib [7]. This patient only responded to high dose of ceftriaxone (150 mg/kg/day) rather than conventional dose (100 mg/kg/day).

Our case of meningitis due to BLNAR strain of Hib is the first case reported from UAE that demonstrated that ceftriaxone was ineffective in treating this condition and child’s prolonged fever only settled after commencing meropenem.

We initially treated this case in accordance with United Kingdom National Institute for Health and Care Excellence (NICE) guidelines on bacterial meningitis (as per our hospital policy) [8]. We used ceftriaxone and dexamethasone early after presentation of child. Unfortunately, fever got prolonged, so we had to change antibiotic to meropenem (according to evidence in literature) as no guidance was available in NICE regarding this.

## Conclusion and take-away messages

We have learned the following important lessons from this interesting case:

- Atypical presentation of acute bacterial meningitis should always be kept in mind. It can mimic mild viral illness of upper/lower respiratory and gastrointestinal tract (common cold, cough, vomiting, or diarrhea).
- It is vital to review antibiotics as per clinical decision supported by culture and sensitivity report.
- There is an excellent evidence of early use (as soon as possible or within 24 hours) of dexamethasone in *Haemophilus influenzae* meningitis to prevent deafness [8].
- It is important to judge each case individually before deciding to perform LP, especially on a sick child, no matter how important a role LP would play in decision-making and management of the child.
- Multidisciplinary team approach is very useful in life-threatening cases like this as it significantly improves chances of survival with minimal or no sequelae.

## Patient perspective

I am father of the patient discussed in this case report. I am very happy that my child has made full recovery from a life-threatening disease without any complications. I have realized that my decision of not completing his vaccination was almost costing his life. I will now educate parents in my family as well as outside about importance of completing immunisation schedule of their children in a timely manner.

## Data Availability

All data generated or analyzed during this study are included in this published article.

## Abbreviations

Hib: *Haemophilus influenzae*
BLNAR: Beta-lactamase-negative ampicillin-resistant
ED: Emergency department
ICU: Intensive care unit
CSF: Cerebrospinal fluid
UAE: United Arab Emirates
CRT: Capillary refill time
GCS: Glasgow Coma Scale
MIC: Minimum inhibitory concentration
LP: Lumbar puncture
NICE: National Institute of Clinical Excellence

## References

[1] Immunisation guidelines Dubai Health Authority, Department of Public Health and Safety, Health Policy and Strategy Sector. https://www.dha.gov.ae/Documents/HRD/Immunization%20Guidelines.pdf.

[2] Dash N, Ameen AMS, Sheek Hussein MM, Smego RA Jr. Epidemiology of meningitis at Al-Ain, United Arab Emirates, 2000–2005. Int J Infect Dis. 2007;11:309–12. 10.1016/j.ijid.2006.05.009 16950640

[3] Otuska T, Okugawa T, Kaneko U. Meropenem was effective to the bacterial meningitis due to beta-lactamase negative ampicillin-resistant Haemophilus influenzae; case report. Jpn J Antibiot. 2002;55(6):861–5. 12621739

[4] Steinberg EA, Overturf GD, Wilkins J, Baraff LJ, Streng. Failure of cefamandole in treatment of meningitis due to Haemophilus influenzae type b; case report. J Infect Dis. 1978;137(Suppl):S180–9. 10.1093/infdis/137.supplement.s180 306403

[5] Mendelman PM, Chaffin DO, Krilov LR, Kalaitzoglou G, Serfass DA, Onay O, Wiley EA, Overturf GD, Rubin LG. Cefuroxime treatment failure of nontypeable Haemophilus influenzae meningitis associated with alteration of penicillin-binding proteins, case report. J Infect Dis. 1990;162(5):1118–23. 10.1093/infdis/162.5.1118 2230238

[6] Abe K, Hoshino T, Imuta N, Nishi J, Ishiwada N. Bacterial meningitis caused by beta-lactamase-negative, ampicillin-resistant nontypeable Haemophilus influenzae in a 1-year-old girl: case report. Kansenshogaku Zasshi. 2014;88(3):291–6. 10.11150/kansenshogakuzasshi.88.291 24974453

[7] Sudo F, Nakamura A, Hoshino T, Ishiwada N, Kohno Y. Successful treatment of beta-lactamase-negative ampicillin-resistant Haemophilus influenzae type b meningitis with high dose ceftriaxone administration. Kansenshogaku Zasshi. 2004;78(7):604–8. 10.11150/kansenshogakuzasshi1970.78.604 15359893

[8] National Institute for Health and Care Excellence (NICE) guidance for meningitis (bacterial) and meningococcal septicaemia in under 16s: recognition, diagnosis and management. Clinical guideline (CG102), Publish date: June 2010, Last updated: February 2015. https://www.nice.org.uk/guidance/cg102/chapter/1-Guidance.

